# A case of coronavirus disease 2019 vaccine‐related myocarditis with late gadolinium enhancement on cardiac magnetic resonance imaging persisting over acute phase

**DOI:** 10.1111/ped.15291

**Published:** 2022-09-08

**Authors:** Ai Konno, Yosuke Osada, Kodai Watanabe, Kenzo Sakurai, Kentaro Aso

**Affiliations:** ^1^ Department of Pediatrics St. Marianna University Kawasaki Japan

**Keywords:** cardiac MRI, COVID‐19 vaccine, gadolinium enhancement, myocarditis, prognosis

The debate over myocarditis following COVID‐19 vaccination is currently a trending topic in medicine. According to the US Centers for Disease Control and Prevention, the frequency of myocarditis/pericarditis following COVID‐19 vaccination is low (12.6 per million doses), and the prognosis is considered good with rapid symptom recovery.^1^ Here we report the case of a 15‐year‐old adolescent male diagnosed with myocarditis after COVID‐19 vaccination who subsequently underwent multiple cardiac magnetic resonance imaging (cMRI) studies, which showed positive late gadolinium enhancement (LGE) even after the acute phase. Three days after the second dose of a COVID‐19 vaccine (BNT162b2) was administered, the patient complained of chest discomfort early in the morning. He visited a local clinic that morning where an electrocardiogram (ECG) was performed, which showed ST‐segment elevation in the II, III, and V3‐6 inductions. Although the chest discomfort resolved and his symptoms lessened over time, he was referred to our hospital the following day for further evaluation. Based on his clinical symptoms and course, a diagnosis of myocarditis caused by COVID‐19 vaccination was made, and he was admitted to our hospital. At the time of admission, his height was 170.2 cm, weight 56.2 kg, blood pressure 112/68 mmHg, heart rate 64 beats per minute (bpm), respiratory rate 20/min, oxygen saturation (SpO_2_) 98% (room air), and body temperature 36.6 °C. Blood examination revealed markedly elevated troponin T (0.351 ng/mL) and mildly elevated N‐terminal pro‐B‐type natriuretic peptide (NT‐pro BNP, 127 pg/mL) and C‐reactive protein (CRP, 0.92 mg/dL), white blood cell 3800/μL, eosinophil 1.7%, hemoglobin 13.8 g/dL, and platelets 152 000/μL. A standard 12‐lead ECG showed ST‐segment elevation at V4–5.

Echocardiography showed normal left ventricular contraction with an ejection fraction of 62% and no clinically problematic atrioventricular valve insufficiency. Due to mild symptoms and good physical condition, a decision was made to observe the patient's clinical course without therapeutic intervention. On day 6 of hospitalization (6 days after onset), laboratory data normalized to troponin T at 0.008 ng/mL and NT‐proBNP at 31.0 pg/mL. Cardiac MRI showed slight dyskinesis from the basal septum to the inferior wall. Increased signal from the basal to the middle and lateral inferior wall on black blood T2WI 2‐lumen imaging and similarly increased signal in the apical region were also present (Fig. 1). Late gadolinium enhancement showed contrast effects mainly in the middle layer from the cardiac base to the center, from the lateral wall to the inferior wall, and in the inferior septal wall. The native T1 value of the LGE‐positive area was within the normal range (T1 value: 1,246–1,274 ms). Although the findings confirmed by cMRI suggested myocardial edema, his physical condition was good, so he was discharged on the seventh day of hospitalization. We performed additional cMRI scans and exercise ECG 6 and 19 weeks after the onset of myocarditis. Cardiac MRI after 6 and 19 weeks showed the presence of LGE mainly in the middle layer of the inferior wall; however, the contrasted area was reduced (Fig. 1). As with the initial cMRI findings, the native T1 value was within the normal range in the area that was determined to be LGE positive. The area that was depicted in white in the T2‐weighted image of the initial cMRI was also no longer observed. Cine MRI showed no dyskinesis and good left ventricle contraction with an ejection fraction of 65%. This case demonstrates that abnormal findings from cMRI can persist in post‐COVID‐19 vaccination myocarditis even after symptoms have abated. The patient's exercise ECG showed no abnormal findings.

**Fig. 1:**
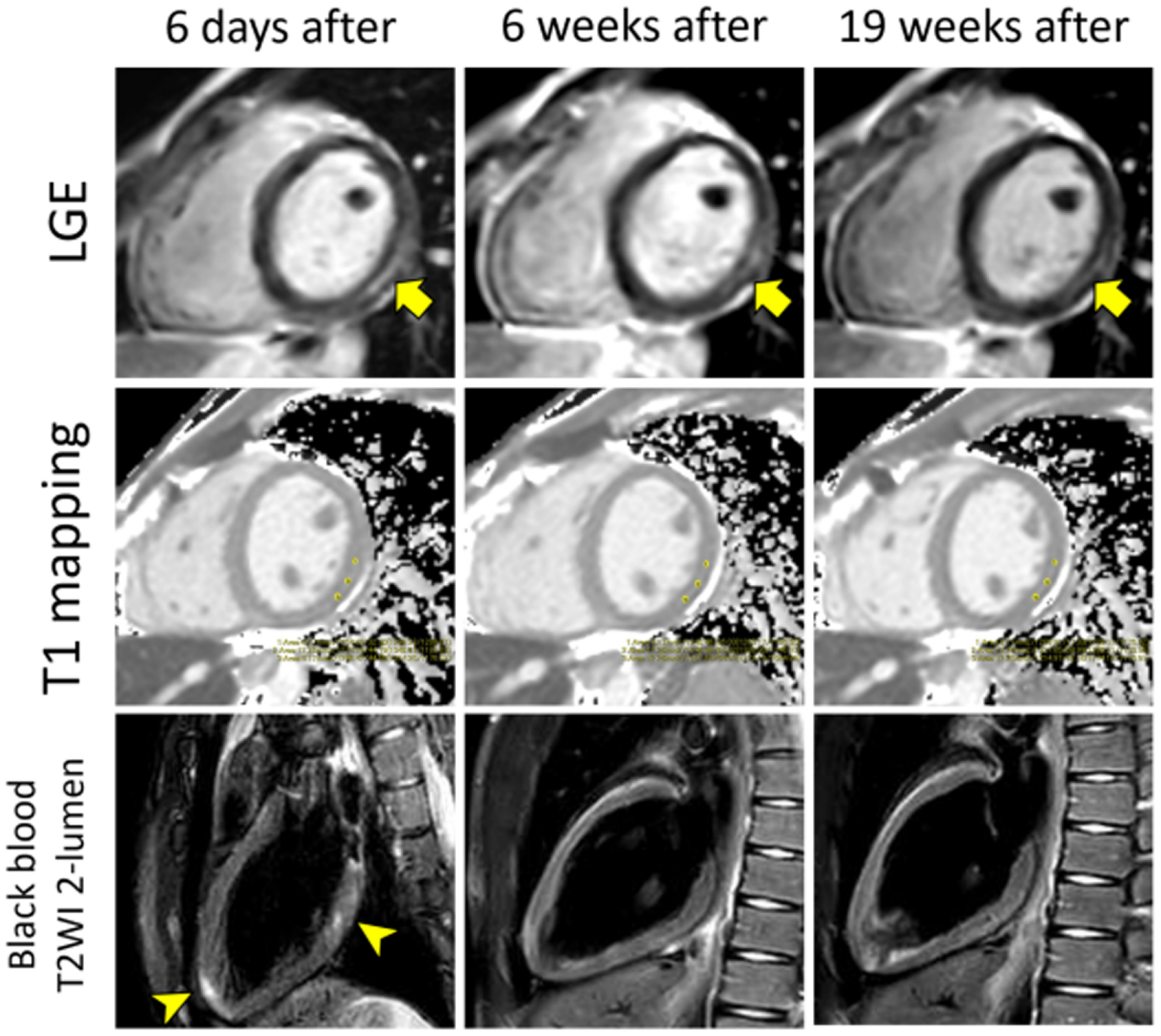
Cardiac MRI imaging at short axis from 6 days after onset (baseline), 6 weeks after onset (second MRI), and 19 weeks after onset (third MRI). Yellow arrows depict myocardial LGE. Native T1 values were within the normal range in the areas determined to be LGE positive. Black blood T2MI 2‐lumen showed areas of high signal intensity in the posterior wall and apex of the left ventricle on initial examination (yellow mark) but there were no abnormal findings on subsequent examinations. LGE, late gadolinium enhancement; MRI, magnetic resonance imaging.

Previous reports have shown a high rate of abnormal MRI findings in post‐COVID‐19 vaccination myocarditis.^2^, ^3^ Recently, several reports have emerged showing residual abnormal findings on cMRI at mid‐term follow up of post‐COVID‐19 vaccination myocarditis, including one report where seven of nine patients had residual abnormal findings at 6‐month follow up.^4^ Positive LGE findings on cMRI in acute myocarditis have been reported to be useful in predicting major cardiovascular events and poor prognosis.^5^

Although post‐COVID‐19 vaccination myocarditis has a favorable prognosis and is considered curable, it may leave abnormalities in the myocardium, as observed in this case; it may therefore be premature to declare it as a complication with a good prognosis.

In the future, the frequency of residual abnormal findings on cMRI and the timing of subsequent follow up should be re‐examined.

## Disclosure

The authors declare no conflict of interest.

## Author contributions

Dr. Konno and Dr. Aso designed the study and drafted the initial manuscript. Dr. Sakurai, Dr. Masumori, Dr. Nakano, and Dr. Osada collected and analyzed the patient data. All the authors have reviewed and revised the manuscript and have approved the final manuscript for submission and agree to be accountable for all aspects of the work.

## Informed consent

The patient's family provided informed consent for the publication of this case report.
